# Recurrent Strokes in a Patient With Metastatic Lung Cancer

**DOI:** 10.7759/cureus.34699

**Published:** 2023-02-06

**Authors:** Rohan Karkra, Riya Jain, Rajendra P Shivaswamy

**Affiliations:** 1 Medicine, JSS (Jagadguru Sri Shivarathreeshwara) Medical College and Hospital, JSSAHER (JSS Academy of Higher Education and Research), Mysuru, IND; 2 Internal Medicine, JSS (Jagadguru Sri Shivarathreeshwara) Medical College and Hospital, JSSAHER (JSS Academy of Higher Education and Research), Mysuru, IND

**Keywords:** chatgpt, recurrent, hemiplegia, stroke, metastasis, lung cancer

## Abstract

A stroke, also known as cerebrovascular accident (CVA), is a medical condition that occurs when the blood supply to the brain is interrupted, resulting in brain cell death. Cancer-associated stroke (CAS) is a rare but serious complication of cancer, where a malignant tumor or its metastases invade or compress the blood vessels in the brain, resulting in a stroke. We describe a case of a 60-year-old male patient recently diagnosed with lung cancer with metastasis to the liver, esophagus, small intestine, and pancreas who has had at least three CVAs within a span of three months. He developed sequelae such as hemiplegia and aphasia. He was managed with dual antiplatelet therapy, neuroprotective drugs, and physiotherapy. Patients with advanced cancers should be routinely screened for neurovascular complications and risks. Prophylactic therapy should be started wherever warranted.

## Introduction

A stroke, also known as cerebrovascular accident (CVA), is a medical condition that occurs when the blood supply to the brain is interrupted, resulting in brain cell death. This can be due to a blocked or ruptured blood vessel, which is referred to as an ischemic stroke or a hemorrhagic stroke, respectively. Stroke is a leading cause of death and disability worldwide, and it can have a significant impact on a person's physical, cognitive, and emotional well-being [1]. Cancer-associated stroke (CAS) is a rare but serious complication of cancer, where a malignant tumor or its metastases invade or compress the blood vessels in the brain, resulting in a stroke [2]. CAS can also occur as a result of cancer treatments such as chemotherapy or radiation therapy. The incidence of CAS varies depending on the type of cancer and its stage, but it is estimated to occur in approximately 1%-2% of cancer patients. The presentation and management of CAS are often different from that of non-cancer stroke, with a poorer outcome. CAS is usually diagnosed in an advanced stage of malignancy, and it is associated with a higher mortality rate. Underlying cancer and the extent of the stroke are the main determinants of the prognosis for patients with CAS [2].

In this case report, we describe a case of a patient with metastatic lung cancer presenting with recurrent stroke. Metastatic lung cancer, also known as stage IV lung cancer, is a serious and advanced stage of the disease in which cancer cells have spread from the lungs to other parts of the body. It is a leading cause of cancer-related deaths worldwide, and the median survival is less than one year.

## Case presentation

A 60-year-old male patient, who is a known case of hypertension, presented with complaints of altered sensorium, weakness of the right upper and lower limbs, and speech impairment for four days. He also developed a loss of consciousness for 10 minutes immediately preceding the presentation to the hospital. The patient was apparently well a week back when he was diagnosed with stage IV carcinoma of the right lung (adenocarcinoma) with the help of computed tomography (CT) imaging and CT-guided biopsy with secondary metastasis to the stomach, liver, small intestine, esophagus, and pancreas, suspected due to lesions noted on abdominal CT such as mass in the pancreas and omental thickening. Metastases were confirmed with the help of a positron emission tomography (PET) CT scan. A peripheral blood cytogenetic analysis (liquid biopsy) was sent just a few days before the presentation to our hospital, but the reports were not available. The patient had a history of similar complaints three months back when he had a stroke of the right basal ganglia and right corona radiata. The patient was then started on dual antiplatelet therapy with neuroprotective agents. The patient continued taking these drugs as a prophylactic measure. One month back, the patient presented to the emergency department of another hospital with a loss of consciousness. He was diagnosed with a transient ischemic attack (TIA).

**Figure 1:**
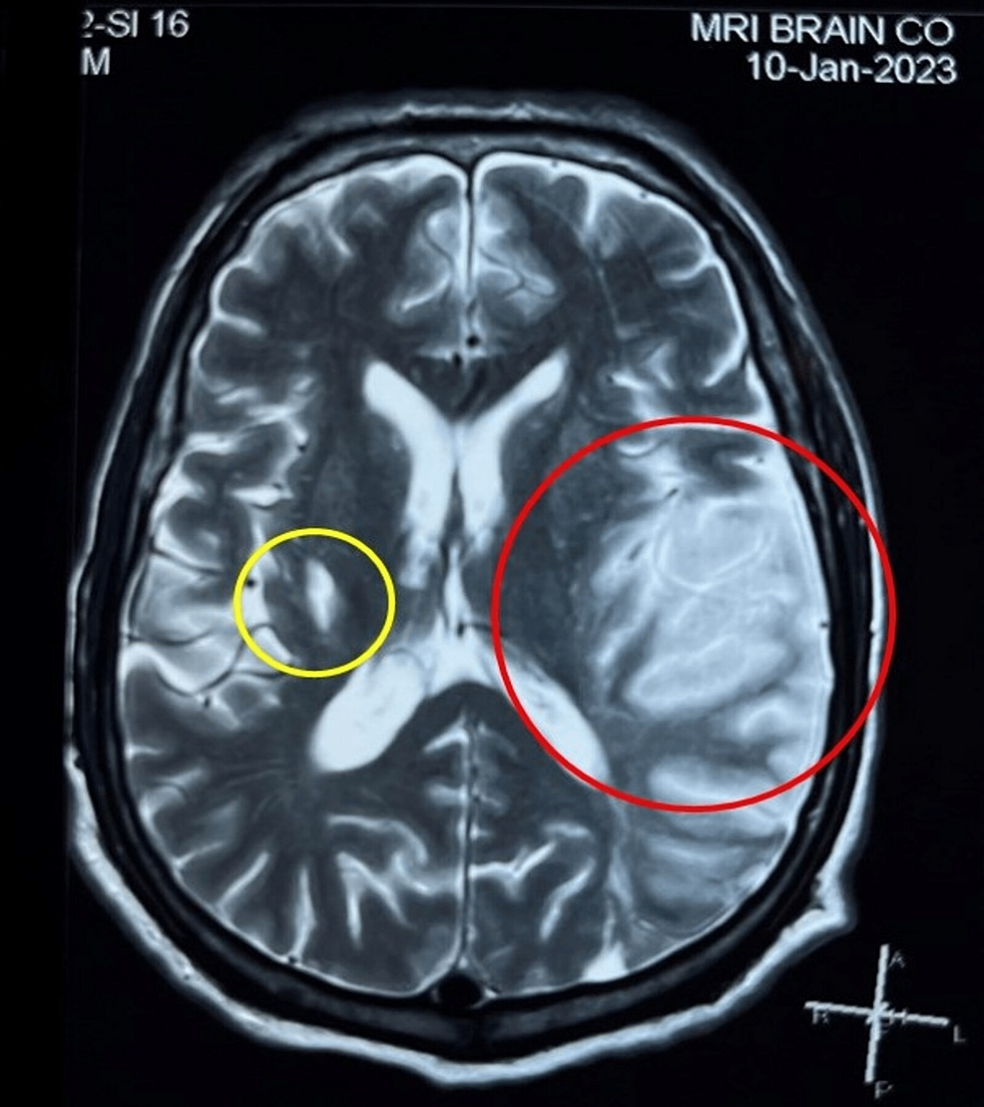
T2-weighted MRI Stroke protocol of the patient shows evidence of acute stroke of the left middle cerebral artery (MCA) territory with chronic infarct in the right ganglion. Red circle indicates acute infarct. Yellow circle indicates chronic infarct.

The patient developed symptoms of limb weakness and slight speech impairment approximately four days before he was brought to the hospital when he lost consciousness for about 10 minutes but regained it by the time he reached the hospital. The patient was not a known smoker or diabetic and had no symptoms suggestive of seizures. On examination, the patient was drowsy (altered sensorium). Hemodynamic vitals were stable. Glasgow Coma Scale (GCS) score was 9 at the presentation. An electrocardiogram (ECG) performed in the emergency department did not identify any rhythm abnormalities. Capillary blood glucose (CBG) levels were normal. A stat MRI scan was ordered as per the hospital stroke protocol. The radiologist reported an acute infarct in the left middle cerebral artery (MCA) territory with chronic infarcts in the left pre-central gyrus and capsule-ganglionic region, right parietal lobe, and caudate nucleus. Chronic lacunar infarcts were also noted in the right frontoparietal lobes. The MRI image is shown in Figure 1.

**Figure 2:**
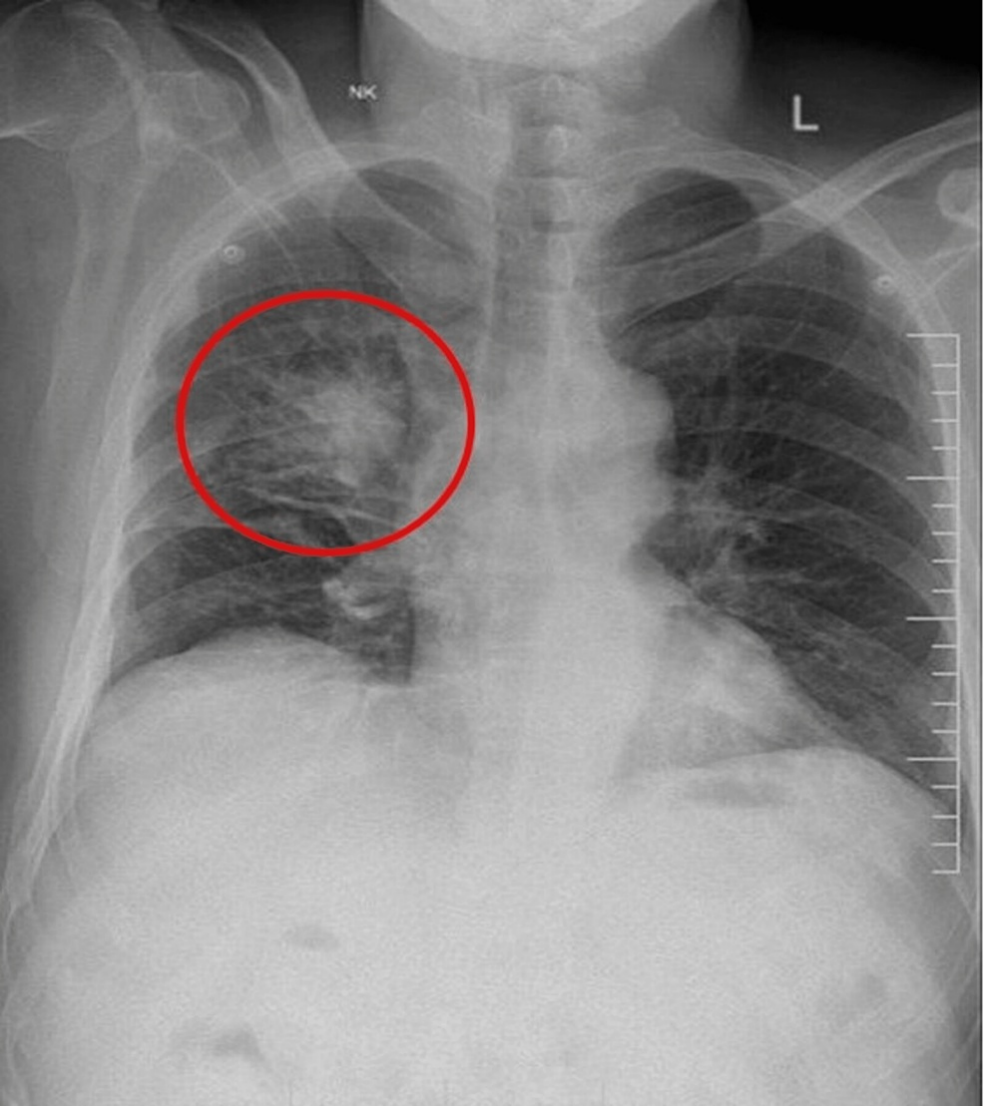
Chest X-ray PA view showing an ill-defined spiculated opacity in the right lung, malignancy (red circle) PA: posterior anterior.

Once the patient's consciousness improved, the patient was found to have Wernicke’s aphasia, and on motor examination, the patient was found to have right-sided hemiplegia with no sensory loss (power was 0/5 in the right upper limb and 2/5 in right lower limb). The patient also had a right-sided upper motor neuron (UMN)-type facial nerve palsy and upgoing plantar reflex (positive Babinski sign). Carotid artery Doppler revealed mild bilateral atherosclerotic disease. A chest X-ray was ordered which showed an ill-defined patchy homogenous spiculated opacity in the upper zone of the right lung field abutting the right horizontal fissure as shown in Figure 2.

Routine investigations of the patients were normal except for non-specific leukocytosis and erythrocyte sedimentation rate (ESR) of 40 mm/hr. The D-dimer level was elevated. The lipid and coagulation profile of the patient was also normal. Two-dimensional (2D) echocardiography ruled out a cardiac cause for an embolus. The patient had already been on dual antiplatelet therapy, aspirin and clopidogrel, thiamine, and statins. Anti-platelet drugs were stopped, and heparin was added to the treatment of the patient. Physiotherapy was also provided to the patient. Unfortunately, the patient passed away two weeks later.

## Discussion

We have described the case of a patient who had three cerebrovascular episodes, i.e., two strokes and one TIA within a span of three months, with his second stroke, unfortunately proving fatal. The patient had advanced metastatic lung cancer which was diagnosed extremely late, and the reason for the delay in diagnosis is yet to be understood.

Cancer-associated stroke (CAS) is a serious complication of cancer, and it has been estimated that one in every seven to eight patients has hidden cancer [3] and one in every 10 has comorbid cancer [4]. Patients may directly present with stroke as the first sign of cancer. Therefore, it is imperative that clinicians are aware of this occurrence and perform reasonable investigations to rule out malignancy in patients with stroke, particularly those with recurrent strokes as in our case. A study by Chen et al. [5] found rates of ischemic stroke to be 1.5 times higher in patients with lung cancer. Xie et al. [6] showed that in patients with lung cancer and stroke, the brain lesions tend to be multiple and have higher d-Dimer, CA125, and CA199 levels.

The mechanism of stroke remains inconclusive, and there are several explanations including hypercoagulability, embolism, accelerated atherosclerosis, hyperinflammatory state, shared comorbidities, risk factors like smoking, and chemotherapy [7]. Chemotherapeutic drugs like vascular endothelial growth factor (VEGF) inhibitors and platinum-based agents have been found to be capable of promoting a procoagulant state within the body which can lead to strokes [8,9].

**Figure 3:**
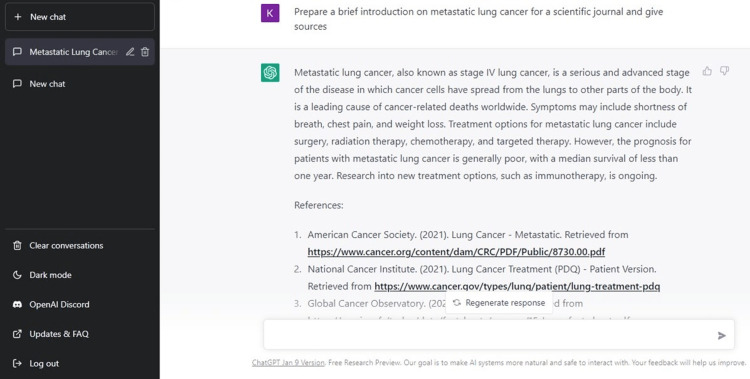
ChatGPT used to write the introduction on metastatic lung cancer

**Figure 4:**
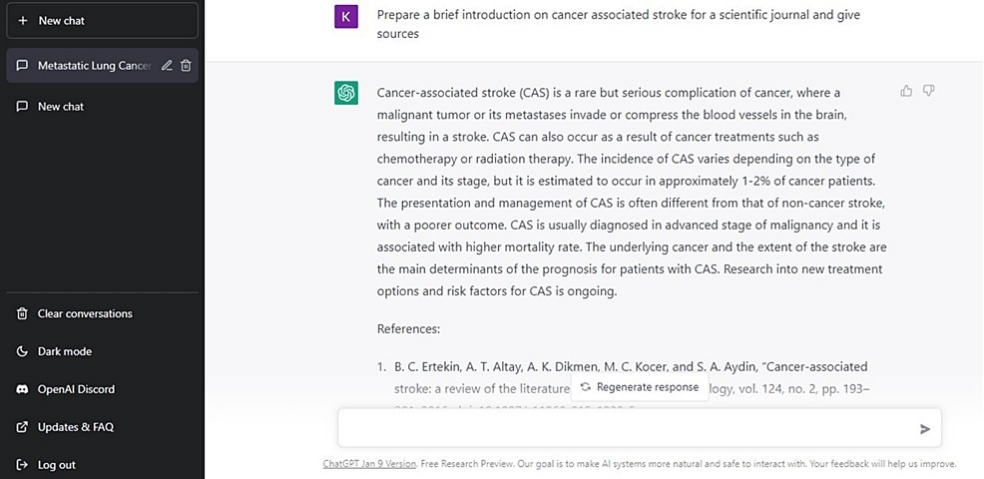
ChatGPT used to write the introduction on cancer-associated stroke (CAS)

There are no defined regulations for the management of CAS, and therefore, it is managed as any other ischemic stroke. While there is evidence of the benefit of thrombolysis, bleeding is almost inevitable. Different hospitals follow different protocols, but mechanical thrombectomy is considered first-line treatment where possible, like in anterior large vessel stroke [10]. Drugs like aspirin and rivaroxaban have also been studied and routinely used in the management of such patients. Neuroprotective drugs like citicoline and edaravone have been shown to improve functional outcomes in patients with ischemic stroke [11]. Experts recommend the use of anticoagulants for the prophylaxis of thromboembolisms in stroke patients. Earlier, heparin was the preferred drug, but recent guidelines have seen a shift toward novel oral anticoagulants (NOACs) [12,13]. Therefore, all patients presenting with cerebrovascular incidents, especially when frequent, should be screened for age and risk-appropriate cancers and offered prophylactic therapy consistent with local guidelines. The introduction to this article was written with the help of ChatGPT as depicted in Figures 3, 4.

## Conclusions

Cancer-associated stroke (CAS) is a rare but serious complication that affects patients with advanced malignancies. Clinicians should be aware of this condition and routinely examine patients for signs of neurovascular complications. Patients and their families should receive adequate counseling, and prophylactic therapy should be provided where needed. Once patients develop CVAs, they should receive appropriate follow-ups, anticoagulant drugs like NOACs, neuroprotective drugs, and physiotherapy.
